# Tanezumab for Patients with Osteoarthritis of the Knee: A Meta-Analysis

**DOI:** 10.1371/journal.pone.0157105

**Published:** 2016-06-13

**Authors:** Shun-Li Kan, Yan Li, Guang-Zhi Ning, Zhi-Fang Yuan, Ling-Xiao Chen, Ming-Chao Bi, Jing-Cheng Sun, Shi-Qing Feng

**Affiliations:** 1 Department of Orthopaedics, Tianjin Medical University General Hospital, 154 Anshan Road, Heping District, Tianjin, 300052, China; 2 School of Nursing, Tianjin Medical University, 22 Qixiangtai Road, Heping District, Tianjin, 300070, China; 3 Department of Ophthalmology, The First Hospital of Jilin University, 71 Xinmin Street, Changchun, 130021, China; Mayo Clinic Minnesota, UNITED STATES

## Abstract

**Objective:**

Tanezumab is a new therapeutic intervention for patients with osteoarthritis (OA) of the knee. We performed the present meta-analysis to appraise the efficacy and safety of tanezumab for patients with knee OA.

**Methods:**

We systematically searched randomized controlled trials from PubMed, EMBASE, and the Cochrane Central Register of Controlled Trials (CENTRAL). The primary outcomes were mean change in the Western Ontario and McMaster Universities Osteoarthritis Index (WOMAC) pain, the WOMAC physical function and patient's global assessment (PGA). Outcomes were reported as the standard mean difference (SMD) or relative risk (RR) with 95% confidence interval (CI). We assessed the pooled data using a random-effects model.

**Results:**

Of the identified studies, four were eligible and were included in this meta-analysis (N = 1839 participants). Compared with the placebo groups, tanezumab yielded a significant reduction in mean change in the WOMAC pain (SMD = 0.51, 95% CI 0.34 to 0.69, P<0.00001), the WOMAC physical function (SMD = 0.56, 95% CI 0.38 to 0.74, P<0.00001) and PGA (SMD = 0.34, 95% CI 0.22 to 0.47, P<0.00001). There was no significant difference in serious adverse events (RR = 1.06, 95% CI 0.59 to 1.92, P = 0.84) between the tanezumab and placebo groups. Tanezumab significantly increased discontinuations due to adverse events (RR = 2.89, 95% CI 1.59 to 5.26, P = 0.0005), abnormal peripheral sensations (RR = 3.14, 95% CI 2.12 to 4.66, P<0.00001), and peripheral neuropathy (RR = 6.05, 95% CI 2.32 to 15.81, P = 0.0002).

**Conclusion:**

Tanezumab can alleviate pain and improve function for patients with OA of the knee. However, considering the limited number of studies, this conclusion should be interpreted cautiously and more clinical randomized controlled trials are needed to verify the efficacy and safety of tanezumab for OA of the knee.

## Introduction

Osteoarthritis (OA) of the knee is the most common location of OA[1], which causes pain, limits activity, and leads to a decreased quality of life[2, 3]. It was estimated that the global prevalence of OA of the knee was 3.8% in 2010[4], and this number will further increase as the elderly population rises. Paracetamol and non-steroidal anti-inflammatory drugs (NSAIDs) are recommended as the first line treatment drugs for painful knee OA[5]. Although patients experience a greater analgesic effect from them over other analgesics, these medications may have a suboptimal therapeutic effect on some patients[6, 7], and some patients experience the risk of hepatotoxicity, gastrointestinal toxicity and cardiorenal side effects[2, 8, 9].

Nerve growth factor (NGF), which plays a crucial role in pain modulation, is a new therapeutic target for pain therapy[10, 11]. All experimental and clinical trials indicate that antagonism of NGF may be a feasible therapeutic option for chronic pain[12–16]. Tanezumab, a humanized monoclonal antibody, blocks NGF from activating TrkA receptors on nociceptive neurons[10, 17]. Although recent randomized controlled trials[18–21] have suggested that tanezumab significantly alleviates pain and improves physical function in patients with OA of the knee, the relatively small number of participants have made their conclusions inconclusive. In a previous meta-analysis comparing an anti-NGF antibody treatment with a placebo in patients with OA of the hip or the knee, Schnitzer and colleagues[22] found that tanezumab appeared to be efficacious in improving symptomatic OA. Because that study investigated the efficacy and safety of tanezumab for patients with OA of the hip or the knee, we cannot determine whether tanezumab is certain to have a significant influence on OA of the knee.

Based on the current clinical studies with tanezumab, we tried to pool the results in a meta-analysis. We adhered to the Preferred Reporting Items for Systematic Reviews and Meta- Analysis (PRISMA) guidelines throughout the study[23]. The purpose of this meta-analysis was to study whether tanezumab was associated with (1) greater mean change in the Western Ontario and McMaster Universities Osteoarthritis Index (WOMAC) pain, (2) greater mean change in the WOMAC physical function, (3) greater mean change in the patient's global assessment (PGA), and (4) fewer adverse events for patients with OA of the knee.

## Materials and Methods

### Search Strategy and Study Selection

We systematically searched randomized controlled trials that investigated the use of tanezumab for the treatment of knee OA from PubMed, EMBASE, and the Cochrane Central Register of Controlled Trials (CENTRAL). The most recent literature search was up to July 25, 2015. Search terms included tanezumab and knee osteoarthritis. Boolean operators “AND” and “OR” were utilized to couple these terms. The details of the search strategy are displayed in S1 Table. There were no restrictions regarding language and publication date. We also manually retrieved reference lists from the identified studies and relevant review studies for additional relevant studies. Two investigators independently assessed the titles and abstracts of studies identified by the retrieval. Then, the full text of the remaining studies were reviewed according to the eligibility criteria. Disagreement was settled by referring to a third reviewer.

### Eligibility Criteria

1. Participants: Only studies enrolling adult participants with a diagnosis of knee osteoarthritis according to the American College of Rheumatology criteria[24] and grade 2 or higher based on the Kellgren-Lawrence[25] grading system.
2. Interventions: The intervention in the experimental group was an intravenous administration of tanezumab at any dose and any phase. Studies with participants receiving NSAIDs or other analgesics, except tanezumab, were excluded.
3. Comparisons: The intervention in the control group was a placebo.
4. Outcomes: Mean change in the WOMAC pain, the WOMAC physical function and PGA, discontinuations due to adverse events, incidence of serious adverse events, abnormal peripheral sensations, and peripheral neuropathy were collected as the outcomes.
5. Study design: Only randomized controlled trials were regarded as eligible in our study.

### Data Extraction and Outcome Measures

Two researchers independently abstracted some necessary information. Information concerning the author, publication year, participant characteristics, intervention and comparison, duration of follow-up, sample size, and outcome were recorded. Any discrepancy was resolved by a joint review of the article to reach a consensus.

The primary outcome measures of interest were mean change in the WOMAC pain, the WOMAC physical function and PGA (using any score or scale). The secondary outcome measures comprised discontinuations due to adverse events, incidence of serious adverse events, abnormal peripheral sensations, and peripheral neuropathy.

If the mean, SD or standard error of the mean (SEM) were not attainable in the text of the articles, we extracted values from the diagrams and tables as needed[26]. For a study with numerous intervention groups, we divided the shared intervention group approximately evenly among the comparisons and included each pair-wise comparison separately in the meta-analysis[26]. If the shared intervention group could not be divided evenly, we paired the shared intervention group with the other intervention groups.

### Risk of Bias Assessment

The tool used to appraise the risk of bias of individual studies was in accordance to the Cochrane Handbook for Systematic Reviews of Interventions (version 5.1.0)[26]. Two investigators independently evaluated all of the studies. The fields of assessment included sequence generation (selection bias), allocation sequence concealment (selection bias), blinding of participants and personnel (performance bias), blinding of outcome assessment (detection bias), incomplete outcome data (attrition bias), selective outcome reporting (reporting bias) and other biases (baseline balance and fund). Each of the fields was determined as a low risk of bias, a high risk of bias, or an unclear risk of bias.

### Quality of Evidence Assessment

The quality of the evidence for all the results was evaluated in accordance with the Grading of Recommendations Assessment, Development and Evaluation (GRADE) methodology[27]. In this methodology, evidence for each outcome was evaluated in five domains: risk of bias, inconsistency, indirectness, imprecision and publication bias[27, 28]. Each result was classified as high, moderate, low, or very low. Two investigators performed the evaluation independently. If a consensus was not reached, a third reviewer was consulted. GRADE Pro, version 3.6 was used to construct summary tables.

### Statistical Analysis

For mean change in the WOMAC pain, the WOMAC physical function and PGA, we calculated the standard mean difference (SMD) and 95% confidence interval (CI). For dichotomous outcomes, we calculated the relative risk (RR) and 95% CI. A random-effects model was applied to estimate the pooled outcomes without regarding heterogeneity[29]. We evaluated heterogeneity using the I^2^ statistic, which mirrored the amount of heterogeneity across trials[30]. Heterogeneity was considered to be statistically significant if the I^2^ value was greater than 50%. For changes in the WOMAC pain, the WOMAC physical function, and PGA, subgroup analyses were performed in accordance with the administration frequency (twice versus three times) and the phase of the trial (phase II versus phase III). Furthermore, we implemented sensitivity analyses to verify the robustness of the study results by using a fixed-effects model and removing trials one by one. To detect the publication bias, we utilized Egger’s linear regression test and funnel plots for primary outcomes if the number of the studies was larger than ten[31]. A P value less than 0.05 was regarded as statistically significant. All statistical analyses were conducted using Review Manager, version5.3 (The Nordic Cochrane Centre, The Cochrane Collaboration, Copenhagen, 2014).

## Results

### Study Search

**Fig 1:**
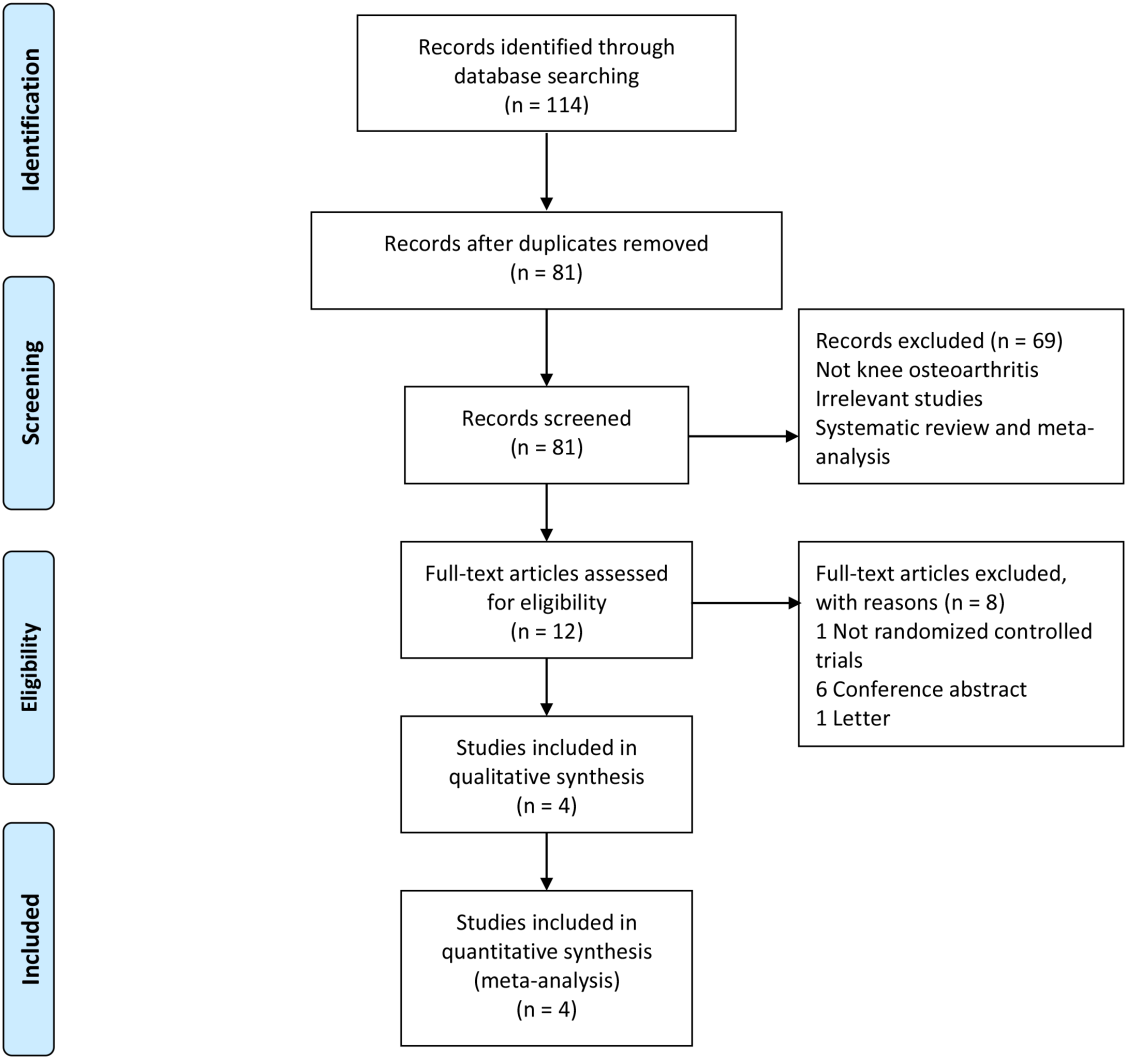
The flowchart of study selection.

The course of study selection is demonstrated in the flowchart (Fig 1). Initially, we identified 114 relevant studies, of which 33 were excluded because of duplicates and 69 did not meet the eligibility criteria at the title and abstract level. After a review of the full text in the remaining 12 studies, one study[16] was excluded for not being a randomized controlled trial, one[32] for being a letter, and six[33–38] for being conference abstracts. Finally, we included 4[18–21] eligible records in the quantitative analysis.

### Study Characteristics

The baseline characteristics of the included randomized controlled trials were outlined in Table 1. There were 4 studies with 15 pair-wise comparison groups included in our meta-analysis. All the studies were sponsored by pharmaceutical companies. There was one article[19] that reported results of two trials; however, one trial did not meet our eligibility criteria because it studied tanezumab for both knee and hip osteoarthritis and, thus, we only used the data from the other trial. Naproxen acted as a control in one study[19]. However, as naproxen did not conform to our inclusion criteria, we discarded the participants treated with naproxen. Two studies[20, 21] were phase II trials, and the other two[18, 19] were phase III trials. Three studies[18–20] were performed in America, and the other one[21] was conducted in Japan. All of the articles were published in English, between 2011 and 2014. The sample size ranged from 6 to 208 (total = 1839).

**Table 1:** Baseline characteristics of studies included in the meta-analysis.

Source	Study characteristics	Phase of trial	Intervention	No. of patients	Mean age (years)	Male (%)	Duration since diagnosis(years)	Follow up
Brown 2012	America.	III	Placebo	172	62.2	30.8	8.2	32 weeks
			Tanezumab 2.5 mg/day	172	60.8	45.3	7.3	
			Tanezumab 5 mg/day	172	62.1	41.3	7.5	
			Tanezumab 10 mg/day	174	61.4	39.1	9.5	
Ekman 2014	America; May, 2009 to August, 2010.	III	Placebo	208	60.9	42.3	9.0	24 weeks
			Tanezumab 5 mg/day	206	61.1	40.8	7.9	
			Tanezumab 10 mg/day	208	61.1	38.5	8.5	
Lane 2010	America; March 30, 2006 to May 3, 2007.	II	Placebo	74	58.1	43.0	NA	16 weeks
			Tanezumab 10 μg/kg	74	58.3	34.0	NA	
			Tanezumab 25 mg/kg	74	59.9	32.0	NA	
			Tanezumab 50 mg/kg	74	60.4	50.0	NA	
			Tanezumab 100 mg/kg	74	57.1	41.0	NA	
			Tanezumab 200 mg/kg	74	58.4	46.0	NA	
Nagashima 2011	Japan; June, 2008 to December, 2009.	II	Placebo	16	59.4	31.3	10.1	13–17 weeks
			Tanezumab 10 μg/kg	15	59.3	33.3	3.8	
			Tanezumab 25 mg/kg	15	57.3	46.7	5.4	
			Tanezumab 50 mg/kg	15	60.7	26.7	5.0	
			Tanezumab 100 mg/kg	16	58.1	25.0	3.1	
			Tanezumab 200 mg/kg	6	60.0	16.7	7.4	

NA: not available.

### Risk of Bias among the Included Studies

**Fig 2:**
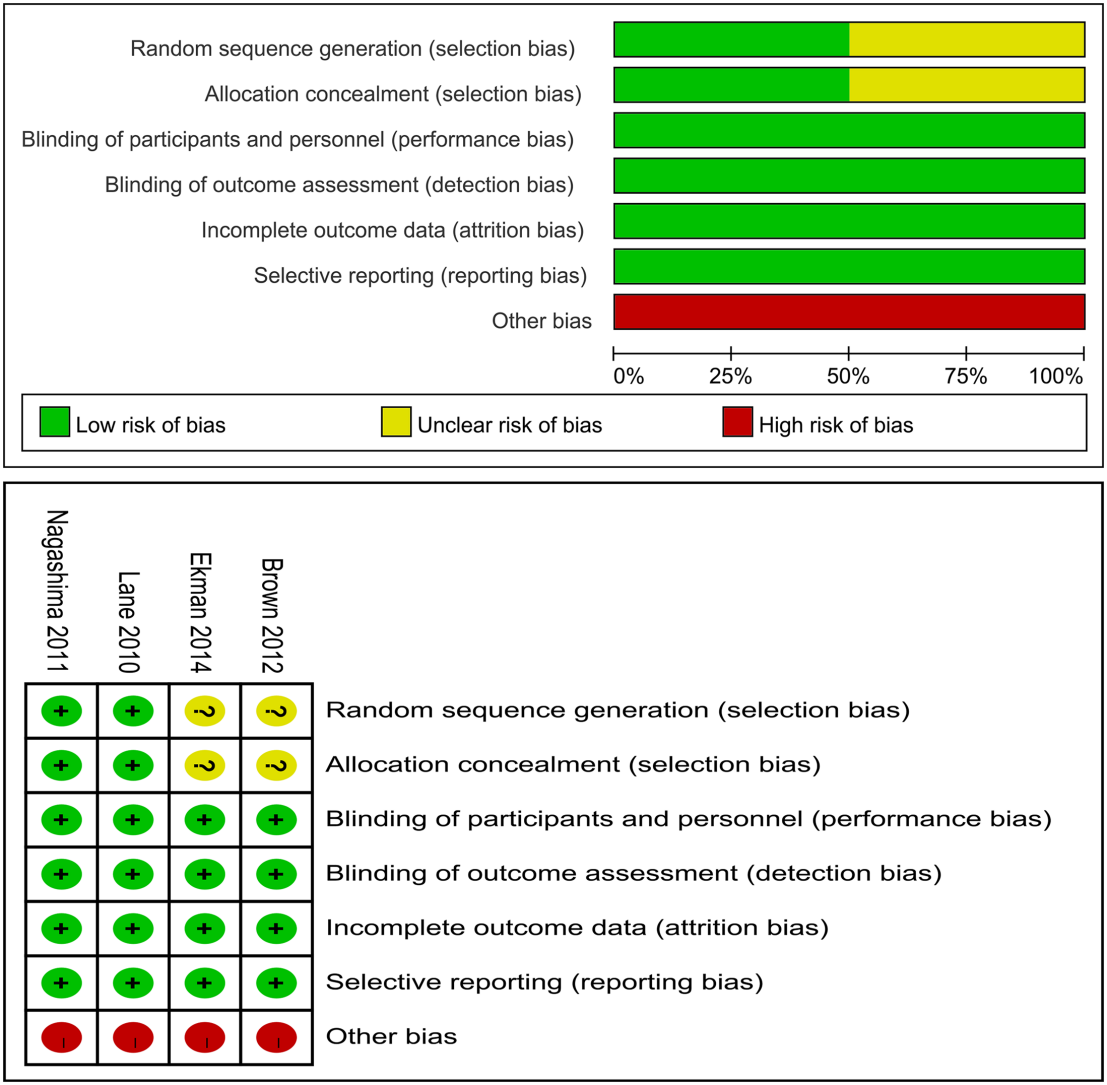
Risk of bias assessment of each included study.

Fig 2 outlines the details of the risk of bias assessment for all of the studies. All of the studies[18–21] were considered to be at high risk of bias. Randomized sequence generation was only implemented adequately in two studies[20, 21], although all of them reported being randomized controlled trials. Allocation concealment was implemented adequately in two studies[20, 21]. All the studies[18–21] reported blinding of the participants, personnel, and outcome assessors. All of the studies[18–21] received funding from companies that produced tanezumab.

### Quality of Evidence Assessment

A summary of the quality of the evidence according to the GRADE approach is shown in Table 2. The GRADE level of evidence was very low for discontinuations due to adverse events; low for the mean change in the WOMAC physical function, serious adverse events, abnormal peripheral sensations and peripheral neuropathy; and moderate for the mean change in the WOMAC pain and mean change in PGA.

**Table 2:** GRADE evidence profile.

Quality assessment							No of patients		Effect		Quality*	Importance
No of studies	Design	Risk of bias	Inconsistency	Indirectness	Imprecision	Other considerations	Tanezumab	Placebo	Relative (95% CI)	Absolute		
**Mean change in the WOMAC pain (Better indicated by lower values)**												
4 studies (15 groups)	randomised trials	serious^1^	no serious inconsistency	no serious indirectness	no serious imprecision	none	1366	467	-	SMD 0.51 lower (0.69 to 0.34 lower)	⊕⊕⊕⊗ MODERATE	CRITICAL
**Mean change in the WOMAC physical function (Better indicated by lower values)**												
4 studies (15 groups)	randomised trials	serious^1^	serious^2^	no serious indirectness	no serious imprecision	none	1366	467	-	SMD 0.56 lower (0.74 to 0.38 lower)	⊗⊗ LOW	CRITICAL
**Mean change in PGA (Better indicated by lower values)**												
2 studies (5 groups)	randomised trials	serious^1^	no serious inconsistency	no serious indirectness	no serious imprecision	none	932	380	-	SMD 0.34 lower (0.47 to 0.22 lower)	⊕⊕⊕⊗ MODERATE	CRITICAL
**Discontinuations due to adverse events**												
3 studies (10 groups)	randomised trials	serious^1^	no serious inconsistency	no serious indirectness	very serious^3^	none	74/1302 (5.7%)	10/750 (1.3%)	RR 2.89 (1.59 to 5.26)	25 more per 1000 (from 8 more to 57 more)	⊕⊗⊗⊗ VERY LOW	IMPORTANT
								0.9%		17 more per 1000 (from 5 more to 38 more)		
**Serious adverse events**												
4 studies (12 groups)	randomised trials	serious^1^	no serious inconsistency	no serious indirectness	serious^4^	none	31/1332 (2.3%)	16/782 (2%)	RR 1.06 (0.59 to 1.92)	1 more per 1000 (from 8 fewer to 19 more)	⊕⊕⊗⊗ LOW	IMPORTANT
								1.4%		1 more per 1000 (from 6 fewer to 13 more)		
**Abnormal peripheral sensations**												
4 studies (15 groups)	randomised trials	serious^1^	no serious inconsistency	no serious indirectness	serious^5^	none	198/1369 (14.5%)	37/830 (4.5%)	RR 3.14 (2.12 to 4.66)	95 more per 1000 (from 50 more to 163 more)	⊕⊕⊗⊗ LOW	IMPORTANT
								4.8%		103 more per 1000 (from 54 more to 176 more)		
**Peripheral neuropathy**												
2 studies (5 groups)	randomised trials	serious^1^	no serious inconsistency	no serious indirectness	serious^6^	none	37/932 (4%)	5/932 (0.5%)	RR 6.05 (2.32 to 15.81)	27 more per 1000 (from 7 more to 79 more)	⊕⊕⊗⊗ LOW	IMPORTANT
								0.6%		30 more per 1000 (from 8 more to 89 more)		

WOMAC = Western Ontario and McMaster Universities Osteoarthritis Index, PGA = patient’s global assessment, SMD = standard mean difference, RR = relative risk.

^1^ All the trials were judged to be at high risk of bias.

^2^ Significant heterogeneity (I^2^ = 52%) was found.

^3^ RR with 95% CI for five trials were 13.00 (0.75–226.68), 7.00 (0.37–133.19), 17.00 (1.00–289.27), 3.00 (0.12–72.47), 9.00 (0.49–164.25), respectively.

^4^ RR with 95% CI for two trials were 3.19 (0.14–72.69) and 3.19 (0.14–72.69).

^5^ RR with 95% CI for one trial was 13.33 (1.93–91.97).

^6^ RR with 95% CI for one trial was 19.00 (2.57–140.63).

*GRADE Working Group grades of evidence: high quality = further research is very unlikely to change our confidence in the estimate of effect; moderate quality = further research is likely to have an important impact on our confidence in the estimate of effect and may change the estimate; low quality = further research is very likely to have an important impact on our confidence in the estimate of effect and is likely to change the estimate; very low quality = we are very uncertain about the estimate.

### Primary Outcomes

#### Mean change in the WOMAC pain

**Fig 3:**
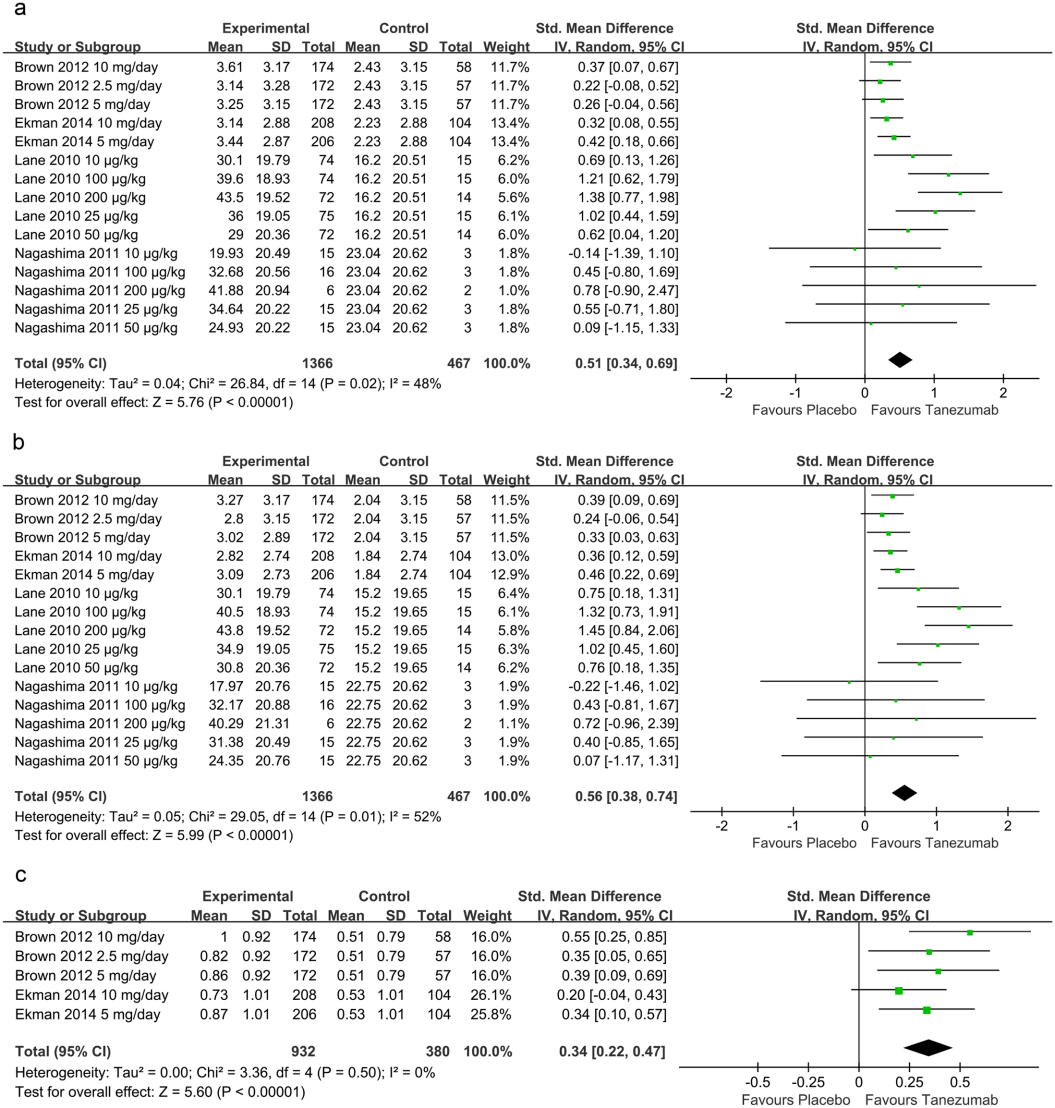
Forest plots of the included studies comparing the mean change in WOMAC Pain (a), WOMAC Physical Function (b), and PGA (c) in patients who received tanezumab and placebo. WOMAC: Western Ontario and McMaster Universities Osteoarthritis Index; PGA: patient's global assessment.

Four studies[18–21] with 15 pair-wise comparison groups, including 1833 patients with knee OA, tested the effect of tanezumab on the mean change in the WOMAC pain. Compared with the placebo, tanezumab was associated with a significant reduction in the mean change in the WOMAC pain (SMD = 0.51, 95% CI 0.34 to 0.69, P<0.00001; I^2^ = 48%) (Fig 3a).

#### Mean change in the WOMAC physical function

Four studies[18–21], including 15 pair-wise comparison groups, reported data from 1833 participants with knee OA and were included in this meta-analysis to estimate the effect of tanezumab on the mean change in the WOMAC physical function. A significant difference was observed in the mean change in the WOMAC physical function between the tanezumab and placebo groups (SMD = 0.56, 95% CI 0.38 to 0.74, P<0.00001; I^2^ = 52%) (Fig 3b).

#### Mean change in PGA

Two trials[18, 19] with five pair-wise comparison groups (1312 participants) were pooled to evaluate the efficacy of tanezumab on the mean change in PGA. Pooled data demonstrated a significant effect that favored tanezumab on the mean change in PGA (SMD = 0.34, 95% CI 0.22 to 0.47, P<0.00001; I^2^ = 0%) (Fig 3c).

### Secondary Outcomes

#### Discontinuations due to adverse events

**Fig 4:**
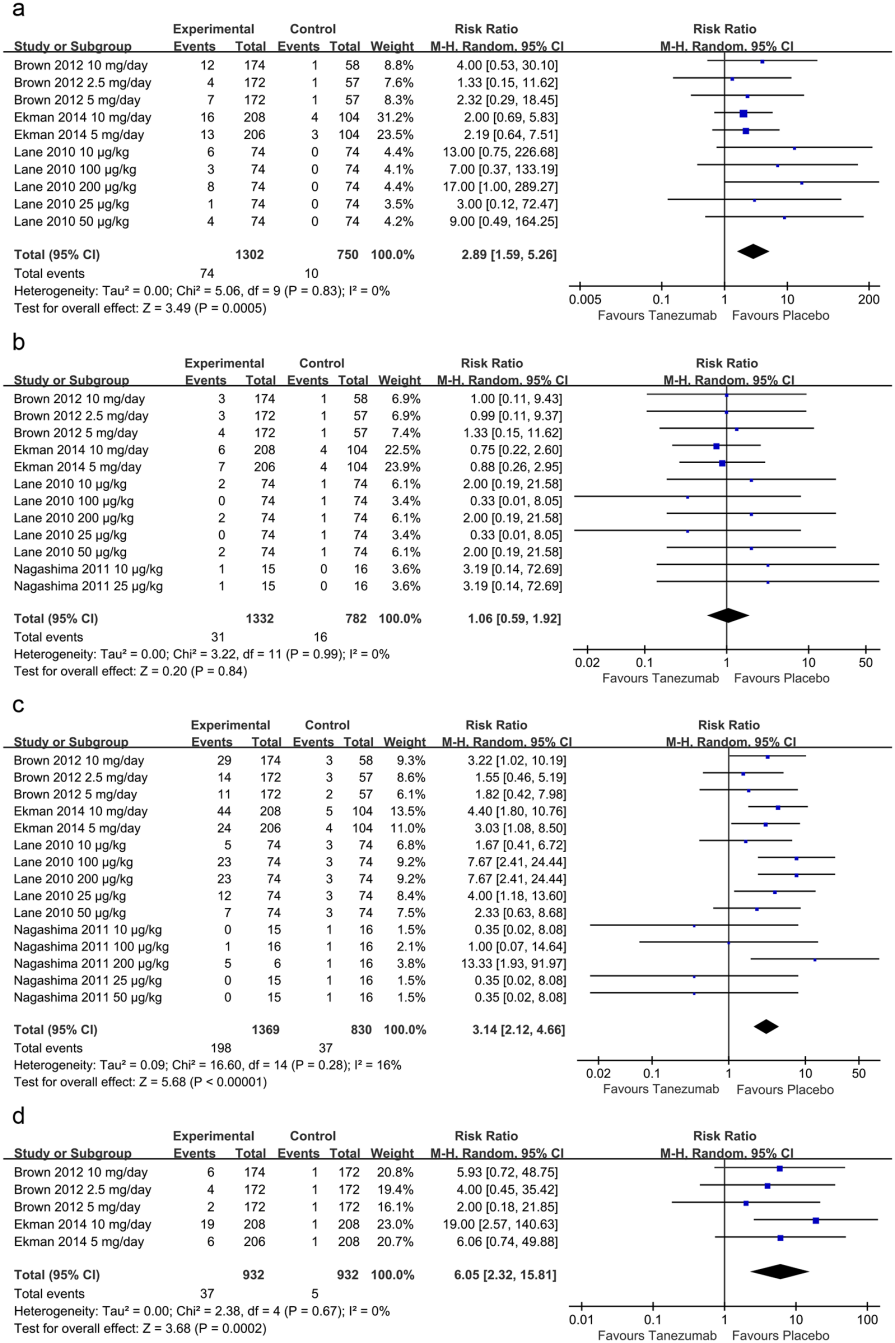
Forest plots of the included studies comparing discontinuations due to adverse events (a), serious adverse events (b), abnormal peripheral sensations (c), and peripheral neuropathy (d) in patients who received tanezumab and placebo.

Three[18–20] studies reported data on discontinuations due to adverse events. Compared with the placebo, tanezumab significantly increased discontinuations due to adverse events (RR = 2.89, 95% CI 1.59 to 5.26, P = 0.0005; I^2^ = 0%) (Fig 4a).

#### Serious adverse events

An adverse event was classified as serious if it was fatal or life-threatening, required or prolonged inpatient hospitalization, was disabling, resulted in a congenital anomaly or birth defect, or required medical or surgical intervention to prevent permanent impairment or damage[20]. Four studies[18–21] with 12 pair-wise comparison groups investigated the number of patients reporting any serious adverse events. There was no significant difference in the number of participants reporting any serious adverse events between the tanezumab and placebo groups (RR = 1.06, 95% CI 0.59 to 1.92, P = 0.84; I^2^ = 0%) (Fig 4b).

#### Abnormal peripheral sensations

A total of four studies[18–21] with 15 pair-wise comparison groups were included in the meta-analysis of abnormal peripheral sensations. Compared with the placebo, tanezumab was associated with a significantly increased incidence of abnormal peripheral sensations (RR = 3.14, 95% CI 2.12 to 4.66, P<0.00001; I^2^ = 16%) (Fig 4c).

#### Peripheral neuropathy

Two studies[18, 19] including five pair-wise comparison groups were included to meta-analyze the incidence of peripheral neuropathy. Compared with the placebo, tanezumab was associated with a significant increase in peripheral neuropathy (RR = 6.05, 95% CI 2.32 to 15.81, P = 0.0002; I^2^ = 0%) (Fig 4d).

### Subgroup Analyses, Sensitivity Analyses and Publication Bias

The subgroup analyses are shown in S1 and S2 Figs for the primary outcome measures. Subgroup analyses indicated that there was no significant difference between the tanezumab and placebo groups in administration frequency (twice versus three times) and phase of trial (phase II versus phase III).

The sensitivity analyses, which involved omitting each study and applying a fixed-effects model, did not alter the outcomes. S2 Table displays the details of the sensitivity analyses.

We were incapable of testing the publication bias because the number of studies was less than ten.

## Discussion

In the current meta-analysis, we evaluated the efficacy and safety of tanezumab for patients with OA of the knee. On the basis of the pooled estimates, tanezumab, compared with the placebo, was associated with a significant reduction in the mean change in the WOMAC pain, the WOMAC physical function and PGA. The use of tanezumab was not associated with a significantly increased risk of serious adverse events, but it increased the odds of discontinuations due to adverse events, abnormal peripheral sensations, and peripheral neuropathy.

The current meta-analysis demonstrated that tanezumab had a beneficial effect on the WOMAC pain, the WOMAC physical function and PGA. In a previous meta-analysis of 13 studies comparing anti-NGF antibody treatment with a placebo in patients with OA of the hip or the knee, Schnitzer and colleagues[22] found that tanezumab appeared to be efficacious in improving the WOMAC pain, the WOMAC physical function and PGA. Although that finding was consistent with our research, that study was intended to investigate the efficacy and safety of tanezumab for patients with OA of the hip or the knee. Thus, we could not determine that tanezumab was certain to have significant influences on the WOMAC pain, the WOMAC physical function and PGA among only patients with knee OA. Therefore, more large scale trials are required to verify the effect of tanezumab on patients with knee OA.

The effect of tanezumab on the WOMAC pain, the WOMAC physical function and PGA was comparable to the roles of the presently recommended NSAIDs or paracetamol[5, 39]. Based on a network meta-analysis[40] of 137 studies in 33,243 adults with knee OA, ibuprofen was associated with a significant reduction in pain and improvement in physical function at 3 months; and diclofenac was associated with a significant decrease in pain and improvement in physical function at 3 months. In a meta-analysis investigating the relative efficacies of NSAID therapies compared with that of a placebo, all NSAIDs were shown to reduce pain[7].

Although both NSAIDs and tanezumab improve pain, tanezumab is different from NSAIDs regarding its effects on pain relief. This may be because tanezumab specifically inhibits the activation of TrkA by NGF[10, 11], rather than blocking the cyclooxygenase pathways[41]. Both experimental and clinical studies have demonstrated that NGF playes a pivotal role in the generation and maintenance of pain[10, 11, 42]. NGF overexpression was found in animal models of experimentally induced osteoarthritis[43]. In humans, there were elevated NGF levels found in the synovial fluid of patients with inflammatory, rheumatoid arthritis or osteoarthritis[42]. Furthermore, inhibition of NGF action remarkably reduced hyperalgesia and pain perception in animal models with acute local inflammation, chronic inflammatory arthritis or osteoarthritis[10, 11, 42, 44].

Regarding the safety of tanezumab, the current meta-analysis showed a significantly increased risk of discontinuations due to adverse events, abnormal peripheral sensations, and peripheral neuropathy. Some discontinuations were thought to be unrelated to the study drug[18]. Additionally, most adverse events were transitory and settled without lasting sequelae within 1 month[18–21]. No significant differences in serious adverse events were found between tanezumab and a placebo. Serious adverse events reported in the studies included appendicitis, bacterial arthritis, cellulitis, spinal stenosis, breast cancer, syncope, inguinal hernia, atrioventricular block, and contusion, although some of them were considered to be irrelevant to tanezumab. However, the most problematic issue is that, the US Food and Drug Administration (FDA) suspended anti-NGF clinical trials in 2010 because of a high incidence of osteonecrosis[45]. Although the FDA lifted their hold on anti-NGF agents on July 19, 2013, there remain some unsolved issues regarding the long-term safety of tanezumab[13]. Therefore, additional non-clinical and clinical studies should be conducted to further confirm the safety of tanezumab[46].

There are some highlights of the present meta-analysis. Our meta-analysis was performed and analyzed in conformity with the best practice methods recommended by the Cochrane Collaboration[26]. A thorough literature search, including PubMed, EMBASE, and CENTRAL, was performed without language restriction. We applied strict and broad inclusion criteria to identify all of the eligible randomized controlled trials in this field. Two investigators independently appraised the risk of bias of the individual studies and assessed the quality of the evidence according to the GRADE approach.

Our meta-analysis also has several potential limitations that should be taken into account when considering the benefits. First, our analysis comprised only four randomized controlled trials, but one of them had a modest sample size (n<100). Compared to large sample size studies, small sample size studies are inclined to overestimate the intervention effect[47], which limits the power of inference. Second, we could not evaluate the potential risk of publication bias due to the small number of included studies, although we deemed our literature search to be exhaustive. Meanwhile, the limited number of studies may also have influenced our conclusions. Furthermore, the follow-up of participants in the included studies was limited. Participants were followed up ranging from 13 to 32 weeks after the initial dose of tanezumab. This may have led to an underestimation of adverse events. Finally, all of the studies were sponsored by pharmaceutical companies. This may also have an influence on the robustness of our conclusions.

## Conclusions

In conclusion, the present meta-analysis demonstrated that tanezumab can alleviate pain and improve function. Furthermore, tanezumab was not associated with a significantly increased incidence of serious adverse events but was associated with significant increases in discontinuations due to adverse events, abnormal peripheral sensations and peripheral neuropathy. Considering the limited number of studies, the conclusion should be interpreted cautiously, and more clinical randomized controlled trials are needed to verify the efficacy and safety of tanezumab for OA of the knee.

## Supporting Information

S1 Fig Forest plots of the mean change in WOMAC Pain (a), WOMAC Physical Function (b), and PGA (c) by subgroup analysis of administration frequency (twice versus three times): tanezumab vs. placebo. WOMAC: Western Ontario and McMaster Universities Osteoarthritis Index; PGA: patient's global assessment.
(TIF)

S2 Fig Forest plots of the mean change in WOMAC Pain (a), WOMAC Physical Function (b), and PGA (c) by subgroup analysis of phase of trial (phase II versus phase III): tanezumab vs. placebo. WOMAC: Western Ontario and McMaster Universities Osteoarthritis Index; PGA: patient's global assessment.
(TIF)

S1 File PRISMA 2009 Checklist. (DOC)

S1 Table Search strategies. (DOCX)

S2 Table Sensitivity analyses. (DOCX)
