# Supplementary figures and images for: Tanezumab for Patients with Osteoarthritis of the Knee: A Meta-Analysis
Source: PLoS One. 2016 Jun 13;11(6):e0157105. doi: 10.1371/journal.pone.0157105 (PMC4905652; doi:10.1371/journal.pone.0157105)

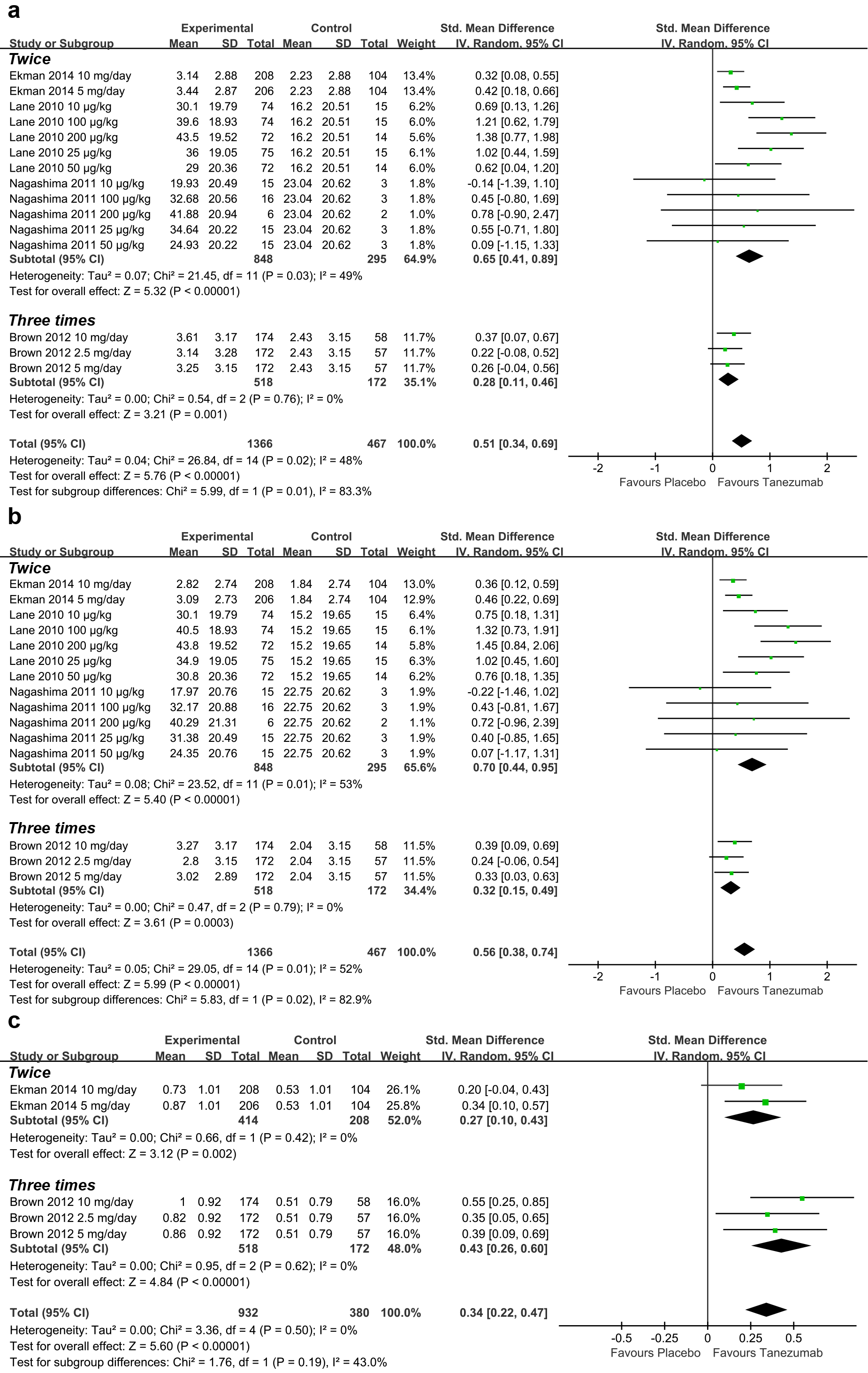

Supplement: S1 Fig — WOMAC: Western Ontario and McMaster Universities Osteoarthritis Index; PGA: patient's global assessment. (TIF) [file pone.0157105.s001.tif]

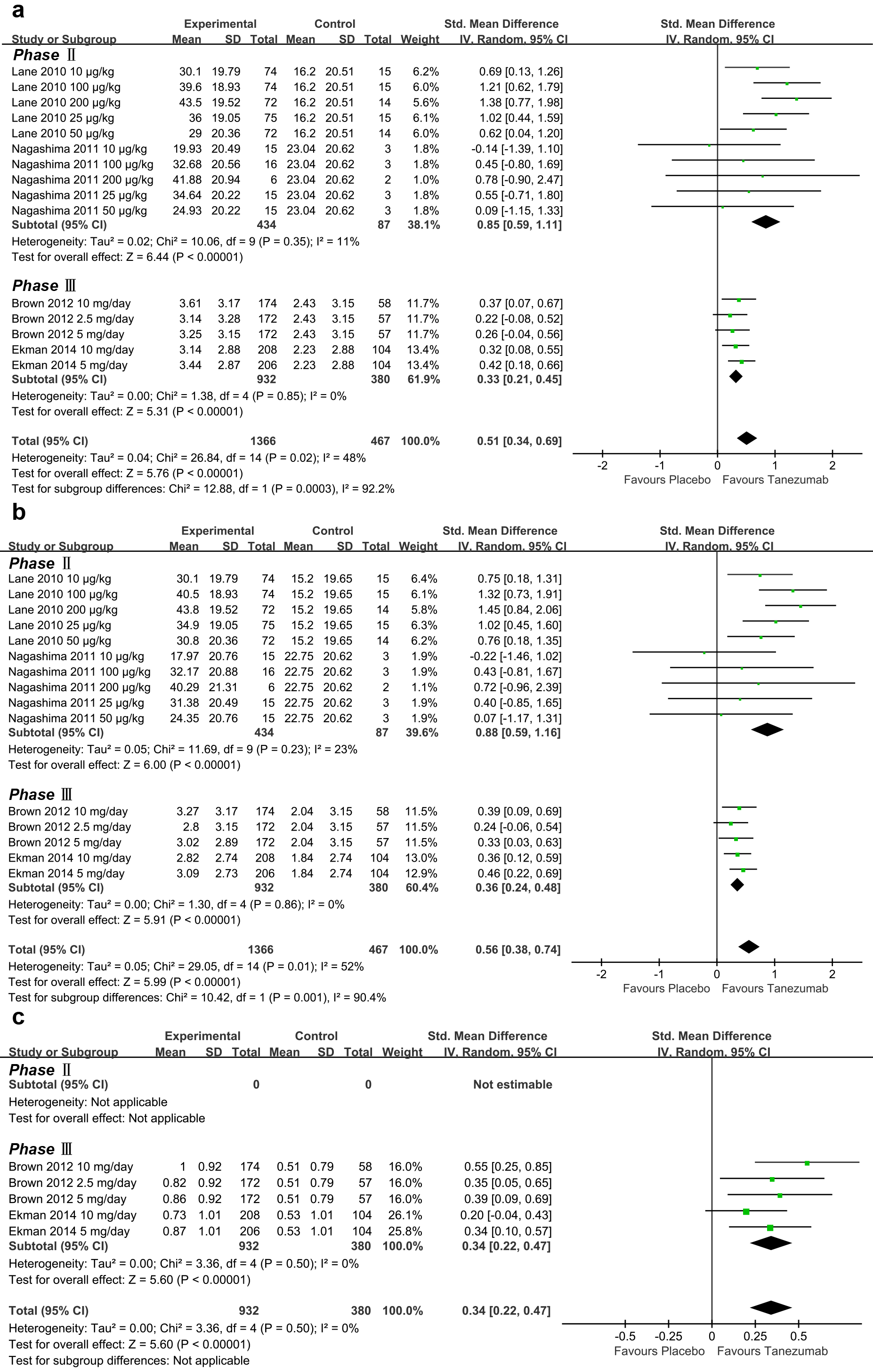

Supplement: S2 Fig — WOMAC: Western Ontario and McMaster Universities Osteoarthritis Index; PGA: patient's global assessment. (TIF) [file pone.0157105.s002.tif]
