# Supplementary material for: Tanezumab for Patients with Osteoarthritis of the Knee: A Meta-Analysis
Source: PLoS One. 2016 Jun 13;11(6):e0157105. doi: 10.1371/journal.pone.0157105 (PMC4905652; doi:10.1371/journal.pone.0157105)
Supplement: S1 Table — (DOCX) [file pone.0157105.s004.docx]

**S1 Table. Search strategies.**

**Source: Pubmed**

**Searched on:** July 25, 15

**Results:** 17

| **Search** | **Query** | **Results** |
| --- | --- | --- |
| #1 | "Osteoarthritis, Knee"[Mesh] | [11747](http://www.ncbi.nlm.nih.gov/pubmed/?cmd=HistorySearch&querykey=2) |
| #2 | knee osteoarthritis[Title/Abstract] | [5483](http://www.ncbi.nlm.nih.gov/pubmed/?cmd=HistorySearch&querykey=3) |
| #3 | #1 OR #2 | [13498](http://www.ncbi.nlm.nih.gov/pubmed/?cmd=HistorySearch&querykey=4) |
| #4 | "Osteoarthritis"[Mesh] | [45592](http://www.ncbi.nlm.nih.gov/pubmed/?cmd=HistorySearch&querykey=6) |
| #5 | (((osteoarthritis[Title/Abstract]) OR osteoarthr*[Title/Abstract]) OR degenerative Arthritis[Title/Abstract]) OR degenerative joint disease[Title/Abstract] | [50572](http://www.ncbi.nlm.nih.gov/pubmed/?cmd=HistorySearch&querykey=7) |
| #6 | #4 OR #5 | [66046](http://www.ncbi.nlm.nih.gov/pubmed/?cmd=HistorySearch&querykey=8) |
| #7 | "Knee Joint"[Mesh] | [44918](http://www.ncbi.nlm.nih.gov/pubmed/?cmd=HistorySearch&querykey=13) |
| #8 | knee*[Title/Abstract] | [104669](http://www.ncbi.nlm.nih.gov/pubmed/?cmd=HistorySearch&querykey=14) |
| #9 | #7 OR #8 | [115918](http://www.ncbi.nlm.nih.gov/pubmed/?cmd=HistorySearch&querykey=15) |
| #10 | #6 AND #9 | [22763](http://www.ncbi.nlm.nih.gov/pubmed/?cmd=HistorySearch&querykey=16) |
| #11 | #3 OR #10 | [23924](http://www.ncbi.nlm.nih.gov/pubmed/?cmd=HistorySearch&querykey=17) |
| #12 | "tanezumab" [Supplementary Concept] | [29](http://www.ncbi.nlm.nih.gov/pubmed/?cmd=HistorySearch&querykey=19) |
| #13 | tanezumab | [55](http://www.ncbi.nlm.nih.gov/pubmed/?cmd=HistorySearch&querykey=22) |
| #14 | #12 OR #13 | [55](http://www.ncbi.nlm.nih.gov/pubmed/?cmd=HistorySearch&querykey=23) |
| #15 | #11 AND #14 | [17](http://www.ncbi.nlm.nih.gov/pubmed/?cmd=HistorySearch&querykey=24) |

**Source: EMBASE**

**Searched on:** July 25, 15

**Results:** 81

| **Search** | **Query** | **Results** |
| --- | --- | --- |
| #1 | 'knee osteoarthritis'/exp | 19566 |
| #2 | 'knee osteoarthritis':ab,ti | 8389 |
| #3 | #1 OR #2 | 20162 |
| #4 | 'osteoarthritis'/exp | 92169 |
| #5 | 'osteoarthritis':ab,ti | 55695 |
| #6 | osteoarthr*:ab,ti | 64139 |
| #7 | 'degenerative arthritis':ab,ti | 1306 |
| #8 | 'degenerative joint disease':ab,ti | 2285 |
| #9 | #4 OR #5 OR #6 OR #7 OR #8 | 106690 |
| #10 | 'knee'/exp | 50209 |
| #11 | knee*:ab,ti | 129913 |
| #12 | #10 OR #11 | 139097 |
| #13 | #9 AND #12 | 31484 |
| #14 | #3 OR #13 | 34490 |
| #15 | 'tanezumab'/exp | 216 |
| #16 | tanezumab | 221 |
| #17 | #15 OR #16 | 221 |
| #18 | #14 AND #17 | 81 |

**Source: Cochrane Central Register of Controlled Trials**

**Searched on:** July 25, 15

**Results:** 16

| **Search** | **Query** | **Results** |
| --- | --- | --- |
| #1 | [Osteoarthritis, Knee][Mesh] | 1390 |
| #2 | knee osteoarthritis:ti,ab,kw | 3776 |
| #3 | #1 OR #2 | 3776 |
| #4 | [Osteoarthritis][Mesh] | 3061 |
| #5 | osteoarthritis:ti,ab,kw OR osteoarthr*:ti,ab,kw OR degenerative arthritis:ti,ab,kw OR degenerative joint disease:ti,ab,kw | 6452 |
| #6 | #4 OR #5 | 6452 |
| #7 | "Knee Joint"[Mesh] | 2107 |
| #8 | knee*:ti,ab,kw | 11506 |
| #9 | #7 OR #8 | 11532 |
| #10 | #6 AND #9 | 3878 |
| #11 | #3 OR #10 | 3878 |
| #12 | tanezumab | 30 |
| #13 | #11 AND #12 | 16 |
