# Supplementary material for: Tanezumab for Patients with Osteoarthritis of the Knee: A Meta-Analysis
Source: PLoS One. 2016 Jun 13;11(6):e0157105. doi: 10.1371/journal.pone.0157105 (PMC4905652; doi:10.1371/journal.pone.0157105)
Supplement: S2 Table — (DOCX) [file pone.0157105.s005.docx]

**S2 Table. Sensitivity analyses.**

| Sensitivity analysis | Heterogeneity | | Effect | |
| --- | --- | --- | --- | --- |
|  | I^2^ | Chi^2^ | SMD and 95%CI | P |
| **Mean change in WOMAC Pain** |  |  |  |  |
| All studies (random model) | 48% | 26.84 | 0.51 (0.34, 0.69) | <0.00001 |
| All studies (fixed model) | 48% | 26.84 | 0.44 (0.33, 0.54) | <0.00001 |
| Exclude Brown 2012 10 mg/day | 51% | 26.63 | 0.54 (0.34, 0.74) | <0.00001 |
| Exclude Brown 2012 2.5 mg/day | 47% | 24.52 | 0.56 (0.37, 0.75) | <0.00001 |
| Exclude Brown 2012 5 mg/day | 49% | 25.32 | 0.55 (0.36, 0.75) | <0.00001 |
| Exclude Ekman 2014 10 mg/day | 49% | 25.58 | 0.55 (0.35, 0.75) | <0.00001 |
| Exclude Ekman 2014 5 mg/day | 52% | 26.82 | 0.54 (0.34, 0.75) | <0.00001 |
| Exclude Lane 2010 10 μg/kg | 50% | 26.01 | 0.51 (0.32, 0.69) | <0.00001 |
| Exclude Lane 2010 100 μg/kg | 35% | 19.87 | 0.45 (0.30, 0.61) | <0.00001 |
| Exclude Lane 2010 200 μg/kg | 25% | 17.43 | 0.44 (0.29, 0.58) | <0.00001 |
| Exclude Lane 2010 25 μg/kg | 43% | 22.75 | 0.47 (0.30, 0.64) | <0.00001 |
| Exclude Lane 2010 50 μg/kg | 51% | 26.42 | 0.51 (0.33, 0.70) | <0.00001 |
| Exclude Nagashima 2011 10 μg/kg | 50% | 25.99 | 0.53 (0.35, 0.71) | <0.00001 |
| Exclude Nagashima 2011 100 μg/kg | 52% | 26.84 | 0.52 (0.34, 0.70) | <0.00001 |
| Exclude Nagashima 2011 200 μg/kg | 51% | 26.67 | 0.51 (0.34, 0.69) | <0.00001 |
| Exclude Nagashima 2011 25 μg/kg | 52% | 26.81 | 0.52 (0.34, 0.70) | <0.00001 |
| Exclude Nagashima 2011 50 μg/kg | 51% | 26.53 | 0.53 (0.35, 0.71) | <0.00001 |
|  |  |  |  |  |
| **Mean change in WOMAC Physical Function** |  |  |  |  |
| All studies (random model) | 52% | 29.05 | 0.56 (0.38, 0.74) | <0.00001 |
| All studies (fixed model) | 52% | 29.05 | 0.48 (0.37, 0.58) | <0.00001 |
| Exclude Brown 2012 10 mg/day | 55% | 28.67 | 0.59 (0.38, 0.80) | <0.00001 |
| Exclude Brown 2012 2.5 mg/day | 51% | 26.35 | 0.60 (0.41, 0.80) | <0.00001 |
| Exclude Brown 2012 5 mg/day | 54% | 28.03 | 0.60 (0.39, 0.80) | <0.00001 |
| Exclude Ekman 2014 10 mg/day | 53% | 27.84 | 0.60 (0.39, 0.81) | <0.00001 |
| Exclude Ekman 2014 5 mg/day | 55% | 29.02 | 0.59 (0.37, 0.80) | <0.00001 |
| Exclude Lane 2010 10 μg/kg | 54% | 28.14 | 0.55 (0.36, 0.74) | <0.00001 |
| Exclude Lane 2010 100 μg/kg | 38% | 20.95 | 0.49 (0.33, 0.66) | <0.00001 |
| Exclude Lane 2010 200 μg/kg | 32% | 19.06 | 0.48 (0.33, 0.63) | <0.00001 |
| Exclude Lane 2010 25 μg/kg | 49% | 25.48 | 0.53 (0.34, 0.71) | <0.00001 |
| Exclude Lane 2010 50 μg/kg | 54% | 28.09 | 0.55 (0.36, 0.74) | <0.00001 |
| Exclude Nagashima 2011 10 μg/kg | 53% | 27.84 | 0.58 (0.39, 0.76) | <0.00001 |
| Exclude Nagashima 2011 100 μg/kg | 55% | 29.05 | 0.57 (0.38, 0.76) | <0.00001 |
| Exclude Nagashima 2011 200 μg/kg | 55% | 28.97 | 0.56 (0.37, 0.75) | <0.00001 |
| Exclude Nagashima 2011 25 μg/kg | 55% | 29.04 | 0.57 (0.38, 0.76) | <0.00001 |
| Exclude Nagashima 2011 50 μg/kg | 55% | 28.65 | 0.57 (0.38, 0.76) | <0.00001 |
|  |  |  |  |  |
| **Mean change in PGA** |  |  |  |  |
| All studies (random model) | 0% | 3.36 | 0.34 (0.22, 0.47) | <0.00001 |
| All studies (fixed model) | 0% | 3.36 | 0.34 (0.22, 0.47) | <0.00001 |
| Exclude Brown 2012 10 mg/day | 0% | 1.26 | 0.31 (0.17, 0.44) | <0.00001 |
| Exclude Brown 2012 2.5 mg/day | 11% | 3.36 | 0.35 (0.21, 0.49) | <0.00001 |
| Exclude Brown 2012 5 mg/day | 8% | 3.25 | 0.34 (0.20, 0.47) | <0.00001 |
| Exclude Ekman 2014 10 mg/day | 0% | 1.34 | 0.40 (0.26, 0.54) | <0.00001 |
| Exclude Ekman 2014 5 mg/day | 11% | 3.35 | 0.35 (0.20, 0.50) | <0.00001 |

WOMAC: Western Ontario and McMaster Universities Osteoarthritis Index; PGA: patient's global assessment; SMD: standard mean difference; CI: confidence interval.
